# Single-cell sequencing: promises and challenges for human genetics

**DOI:** 10.1515/medgen-2022-2156

**Published:** 2022-11-29

**Authors:** Varun K. A. Sreenivasan, Jana Henck, Malte Spielmann

**Affiliations:** Institute of Human Genetics, University Hospital Schleswig-Holstein, University of Lübeck and Kiel University, 23562 Lübeck, 24105 Kiel, Germany; Human Molecular Genomics Group, Max Planck Institute for Molecular Genetics, D-14195 Berlin, Germany; DZHK e. V. (German Center for Cardiovascular Research), Partner Site Hamburg/Kiel/Lübeck, 23538 Lübeck, Germany

**Keywords:** single-cell sequencing, disease characterization, phenotyping, diagnostics, therapy, human genetics, cell atlas, saturation gene editing, CRISPR, cellular composition

## Abstract

Over the last decade, single-cell sequencing has transformed many fields. It has enabled the unbiased molecular phenotyping of even whole organisms with unprecedented cellular resolution. In the field of human genetics, where the phenotypic consequences of genetic and epigenetic alterations are of central concern, this transformative technology promises to functionally annotate every region in the human genome and all possible variants within them at a massive scale. In this review aimed at the clinicians in human genetics, we describe the current status of the field of single-cell sequencing and its role for human genetics, including how the technology works as well as how it is being applied to characterize and monitor diseases, to develop human cell atlases, and to annotate the genome.

## Introduction

The advent of next-generation sequencing (NGS) technologies has made the screening of patients and the discovery of new variants routine; however, the task of deciphering the impact of the uncovered genomic alterations is still the central challenge of the field of human genetics. Over 60 % of patients with rare diseases of probable genetic etiology leave the clinic without a diagnosis even after whole-genome sequencing according to the pilot report from the UK 100,000 genomes project [1]. Improving this necessitates multifaceted efforts, starting with thorough characterization of the “normal or wild-type” state of tissues to benchmark diseased states against, obtaining a complete picture of genotype–phenotype relationships across variants, and developing technologies that facilitate clinical translation. Advances in all three areas will be decisive in developing effective diagnosis and therapeutic regimes.

One technology that attracted an enormous amount of attention lately across many fields is single-cell sequencing (sc-seq). While sequencing of tissues (hereafter referred to as bulk-seq) has been a remarkable method to characterize the average profile of a tissue in health and in disease, it becomes insensitive in detecting a phenotype, when a disease affects only a subpopulation of cells in an organ or tissue leading to fraught conclusions. Sc-seq technologies enable acquiring more granular information about the different cell types within a tissue, thus increasing not only the resolution of the data but also the statistical power when benchmarking a diseased state to a normal state. That is, it is theoretically possible to compare sequencing data from patients to a “wild-type” sample to determine gene expression as well as cell composition changes, even at early stages of disease progression. This is just one of the reasons why sc-seq was named the method of the year in 2013 (*Nature Methods*) and breakthrough of the year in 2018 (*Science*), has been highlighted for early detection of diseases [2], and draws over 8700 publications per year (“single-cell” on PubMed). Creation of such “wild-type” benchmarks – the so-called cell atlases – has been one of the major achievements of the field. What cell atlases are and how they can be used is discussed towards the end of this review. Sc-seq as an unbiased and high-resolution phenotyping method has also been paralleled with multiplexed gene editing methods, such as pooled CRISPR and saturation genome editing. This combined genotype–phenotype screening allows annotation of hundreds to thousands of genomic regions in one single experiment, which is also briefly discussed.

This review gives an introduction to the field of single-cell genomics with a specific focus on its use for human geneticists. We describe the current state of sc-seq technology and how it applies to human genetics. First, we briefly outline the experimental and analytical workflows. Then we review sc-seq applications most relevant to the field of human genetics and discuss how the toolbox comprising various flavors of sc-seq is being integrated into the field of medical genetics. In the interest of brevity, we have restricted the scope of the review based on the definition of the technology and the biological focus of the studies. We define sc-seq as technologies that provide cellular resolution, use sequencing as a read-out strategy (cf. hybridization probes), and probe cellular nucleic acids (cf. proteome sequencing). We limit our discussions to studies which focus on human tissues and diseases relevant to human genetics, except for the section on the applications under development. For more focused reviews on these topics, we suggest the following reviews [3], [4], [5], [6].

## Experimental and analytical workflows

**Figure 1:**
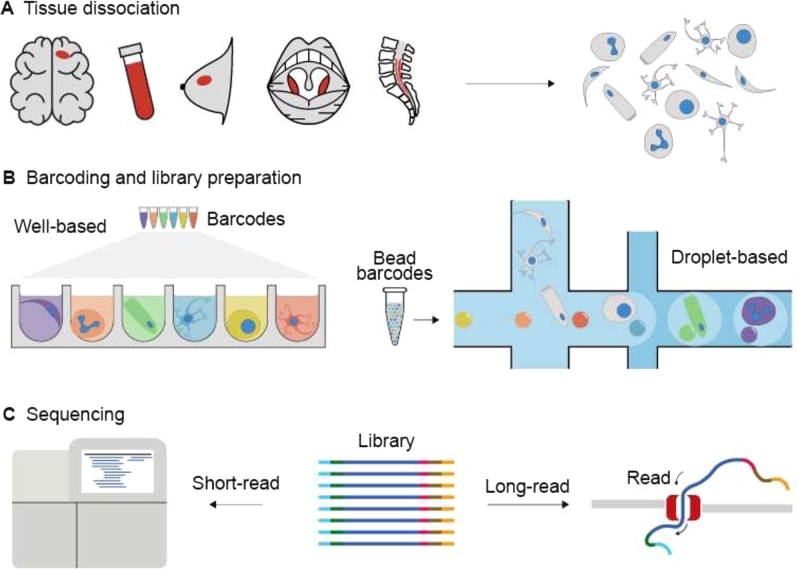
The experimental workflow of sc-seq. **(A)** The workflow starts with the dissociation of the biopsy samples into a cellular or nuclear suspension. **(B)** This is followed by cellular barcoding using droplet- or well-based technologies that enables pooled sequencing of the molecules from all the cells. **(C)** The final step is sequencing. Although long-read sequencing is rarely used in current analysis workflows, it has potential for identification of splice variants in the case of sc-transcriptome-seq or structural variants in the case of sc-genome-seq as well as to reduce sequencing costs [22]. Adapted from [10].

As the name implies, sc-seq comprises the extraction of the nucleic acids of interest from individual cells or nuclei (hereafter collectively referred to as cells for simplicity), followed by sequencing and data analysis (Fig. 1). Here we provide a quick summary of the modalities of sc-seq as well as the experimental and analytical workflows, which have been elaborated and critically evaluated in many recent reviews [4], [7], [8], [9], [10].

In its most basic form, there are three main modalities of sc-seq where the cellular nucleic acids are sequenced – sc-genome-seq, sc-epigenome-seq, and sc-transcriptome-seq. Each of these modalities offer complementary information that are uniquely suited to solve niche challenges in human genetics. Sc-genome-seq is suited for applications such as identifying genotypes (albeit with sparse coverage when compared to bulk-genome-seq), elucidating mechanisms that lead to somatic mutations [11], constructing developmental lineages, and prenatal testing [12]. On the other hand, sc-epigenome and sc-transcriptome offer the possibility to phenotype cells and also elucidate the mechanistic pathways leading from the genotype or environmental cues to a pathological state. More recently, multi-ome or multimodal sequencing has enabled the simultaneous profiling of combinations of these modalities, for example to link regulatory elements to gene expression profiles and to deduce gene regulatory networks [13], [14].

The experimental workflow of sc-seq resembles bulk-seq for the most part, including PCR steps, enzymatic fragmentation, and end-repair, as well as sample-index and sequencing-adapter ligation. However, there are two major steps that differentiate sc-seq from bulk-seq – dissociating the tissue to cells and cellular barcoding (Fig. 1A, B). Dissociating the sample into a single-cell suspension enables access of reagents to individual cells and thereby the extraction of molecules of interest, without mixing the molecular contents between cells. This step requires painstaking optimization, since the tissue type, whether it is fresh or frozen, and the biological age of the sample all affect the protocol. Follow-up flow-assisted cell sorting (FACS) may be required to filter out cell debris. Often nucleus sequencing is preferred over cell sequencing due to the ease of dissociation.

Cellular barcoding is in principle similar to sample indexing – a prevailing method for cost reduction in NGS, where molecules (or sequencing libraries) from multiple samples are multiplexed using unique oligonucleotide indices prior to pooled sequencing (Fig. 1C). The resulting data is bioinformatically demultiplexed based on the known indices. In cellular barcoding, unique oligonucleotides instead demarcate the molecules with cellular identities. But unlike sample indexing, where indices refer directly back to a particular metadata of the sample (e. g., treatment vs. control group, or the experimental replicate #), cellular barcoding occurs at random and the identities of the cells (e. g., cell type) need to be bioinformatically determined. Several technologies have been developed for cellular barcoding. Each employs a different strategy to append oligonucleotide barcodes to molecules within each cell. These include using micro/nano-wells (Fig. 1B) [15], [16] to isolate cells in individual reaction chambers, or microfluidics-based droplets [17], [18] or split-pool methods that use combinatorial methods to achieve this goal without cellular isolation [19]. Kits that simplify most of the steps from cellular barcoding to generation of sequencing libraries are commercially available from BD Rhapsody, 10x Genomics, TakaraBio, MissionBio, and Standard Biotools, among others. A comparison of some of these technologies can be found in several reviews [10], [20], [21].

The resulting sequence datasets are generally large and complex. Their large size is a result of the fact that most recent sc-seq methods readily sequence well over thousands and up to millions of cells [23]. The datasets are challenging because of artifacts arising from the limited amount of nucleic acid molecules within each cell (e. g., dropout events, as discussed in the Technological limitations section). Apart from additional correction and filtering measures to account for the artifacts and that the sequencing reads need to be demultiplexed using the cellular barcodes to generate an omics profile per cell, the main analytical workflow also resembles that of bulk-seq. The genomic features, i. e., variants in sc-genome-seq, gene expression counts in sc-transcriptome-seq, or read counts in sc-epigenome-seq, are then extracted from the raw data by alignment to the reference genome. The extracted features are segregated per cell and tabulated into a cell × feature matrix. At this point in the analytical workflow, the cell names in this matrix are alphanumerals corresponding to the oligonucleotide barcodes and do not contain any interpretable metadata (e. g., cell type or cellular genotype).

**Figure 2:**
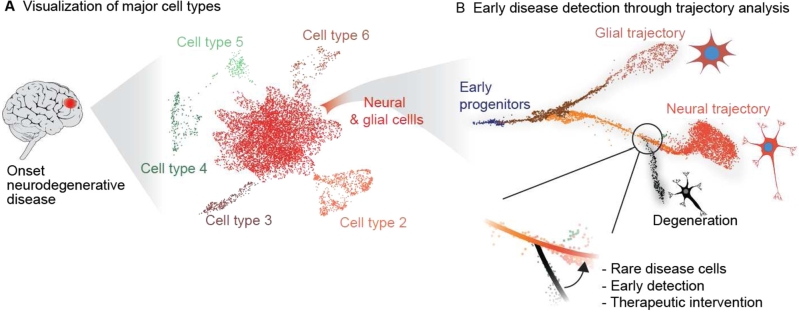
Analysis of sc-seq data for early disease detection. **(A)** The sc-seq data obtained from a brain biopsy can be visualized in the form of a UMAP embedding, where each cell is represented by a dot. Cells (dots) with similar phenotype (transcriptome or epigenetic marks) cluster together, which are assigned to cell types based on prior knowledge. **(B)** Clusters in A might contain cells undergoing transition, such as differentiation or cell cycles, which can be visualized by means of trajectory analysis. Here, cells that deviate from established healthy trajectories may aid in early disease detection. Based on [10] and [2]. Note – synthesized data.

The next steps of the workflow are generally aimed at inferring this metadata from the data by means of (hierarchical) clustering of the cells (Fig. 2A) and the identification of differential features between clusters. Cell type annotation, i. e., the identification of cell type corresponding to each cluster, is usually performed manually. However, thanks to the recent boom in the abundance of public cell atlases, automated methods are also becoming available [24]. However, the accuracy and sensitivity of annotations to smaller and previously undescribed cell populations may still be limited, making manual curation a necessary step. From this point on, the downstream analyses vary significantly based on project goals, such as identifying mutational landscapes and differentially expressed genes, calculating cellular compositions, or establishing gene regulatory networks stratified either by sample groups or by the identified cell clusters. Several bioinformatic methods have been developed to handle all aspects of the analysis. Some of these include Cellranger, Seurat, Scanpy, etc., for sc-transcriptome-seq analysis [25], chromVar, Signac, scABC, etc., for sc-epigenome-seq analysis [26], and Monovar, SCIΦ, etc., for sc-genome-seq analysis [27]. Nevertheless, the sc-seq data continues to be vulnerable to subjective analysis, and is best left to experienced bioinformaticians. Automated and web-based interactive tools are becoming available, which will make sc-seq more accessible to a diagnostics setting [28], [29].

## Applications of sc-seq technologies

In this section, we review the literature on applying sc-seq to human genetics. This section is divided into three parts: (i) applications in disease characterization, (ii) applications to aid diagnosis or therapy, and (iii) applications under active development.

### i. Applications in disease characterization – Cellular phenotyping and deciphering molecular mechanisms

Sc-seq is suited to the characterization of diseases and underpinning the molecular mechanisms behind the pathology. This is especially the case when the pathological state affects a subset of cells (e. g., cell type-specific) or when it affects multiple organs or tissues because of pleiotropic genes. Given the single-cell resolution, it also enables identification of co-occurring mutations from mutually exclusive ones, which is not possible in bulk-seq [12]. These advantages have led to the application of sc-seq for the characterization of various diseases, including infectious diseases such as HIV, tuberculosis, influenza, COVID-19, etc., which have already been reviewed recently [12], [30], [31]. A large proportion of applications on non-infectious diseases focuses on cancer, namely the characterization of cellular heterogeneity, gene pathways, clonal evolution, etc. [32], [33], [34], [35], [36]. Sc-seq has also been used to characterize complex genetic diseases [37] such as heart diseases [38], Diamond–Blackfan anemia [39], and autism [40]; auto-immune conditions such as lupus [41], multiple sclerosis [42], and rheumatoid arthritis [43]; respiratory illnesses such as asthma [44], [45], [46]; and tissue degenerative conditions such as aging [47], [48], age-related ocular diseases [49], [50], [51], Alzheimer’s disease [52], [53], Parkinson’s disease [54], [55], [56], and ALS [57]. These efforts have also resulted in publicly accessible databases similar to wild-type cell atlases, providing an easy portal to query the expression patterns of genes of interest (e. g., scREAD for Alzheimer’s disease [52]). To a lesser extent, sc-seq has also been used to characterize monogenic and chromosomal disorders, which are summarized in Table 1. In order to portray the power of sc-seq for disease characterization and molecular phenotyping of a mutation, we describe two of these studies in Boxes 1 and 2.

**Box 1.**

**Trisomy.** Autosomal polyploidy, such as trisomy 21 and 18, is associated with decreased cellular proliferation, congenital defects, intellectual disability, and shortened life expectancy. In the case of trisomy 21 (Down syndrome), it also leads to impaired memory [58] as well as a higher predisposition for Alzheimer’s disease, with its clinical hallmark of plaques [59]. The etiology of the syndrome is, however, not fully known. Palmer et al. [60] applied sc-transcriptome-seq on cerebral cortices from 29 age-, sex-, and quality-matched Down syndrome and control brains to characterize the differential cellular constitution as well as isoform-specific expression profiles. One of the primary observations in this study was the imbalance between the numbers of inhibitory and excitatory neurons in the cortex – an observation reported previously in mouse models. This imbalance was seen in all examined brains, but limited to the interneurons developing from the caudal (as opposed to medial) ganglionic eminence. As opposed to naive expectation and in congruence with previous investigations, the expression of only nine genes correlated with the polyploidy (i. e., fold expression change > 1.5), and most of the affected genes are not located in chromosome 21. Moreover, the misexpression in the Down syndrome group was cell type-dependent, with microglia being the most affected cell type. Additionally, signatures of aging were found in the microglia from young Down syndrome samples. The Down syndrome microglia also overexpressed components of *C1q* and *ADGRG1*, which are implicated in overactive synapse pruning and in memory loss. In short, the authors identified cellular processes that could mediate the phenotypic consequences of Down syndrome, which would have gone undetected by bulk-transcriptome-seq or other comparable methods.

**Table 1:** A list of sc-seq-based studies to phenotype and elucidate monogenic and chromosomal disorders.

Genetic disorder	Single-cell modality	Goal	Tissue/cell type analyzed	Main findings	Reference
Autosomal dominant polycystic kidney disease (ADPKD)	Transcriptome	Cell composition and origin of ADPKD	Kidney	Cysts can originate from multiple renal tissues with varying gene set activation	[62]
Autosomal dominant polycystic kidney disease (ADPKD)	Transcriptome, chromatin accessibility	Cell type and mechanisms driving ADPKD	Kidney	GPRC5A upregulated in cyst lining cells	[63]
Huntington’s disease (HD)	Transcriptome	Phenotype astrocytes in HD	Cingulate cortex; astrocytes and other cells	HD astrocytes of multiple states, some upregulating and some downregulating key genes compared to the control	[64]
Huntington’s disease (HD)	Transcriptome	Study changes in cerebrovascular cells	Brain; vascular cells	Activation of innate immune signaling in vascular and glial cells, reduction of blood–brain barrier integrity	[65]
Huntington’s disease (HD)	Transcriptome	Study mechanism leading to neuronal death	Brain; striatal neurons	Upregulation of innate immune signaling in spiny projection neurons	[66]
Cystic fibrosis	Transcriptome	Characterize sputum cells	Sputum	Shift of immune cell repertoire, altered phagocytic and cell survival pathways	[67]
Cystic fibrosis	Transcriptome	Characterize disease-related changes to proximal airway	Lung	Increase of epithelial cells transitioning to ciliated and secretory cells	[68]
Systemic sclerosis-associated interstitial lung disease (SSc-ILD)	Transcriptome, multimodal (transcriptome + proteome)	Identify and define fibroblast transcriptome to understand pathogenesis	Lung; fibroblasts	Myofibroblasts undergo greatest phenotypic changes; myofibroblast differentiation and proliferation are key disease mechanisms	[69]
Sickle cell disease (SCD)	Transcriptome	Characterization of CD34tsup+ hematopoietic stem/progenitor cells	Bone marrow; CD34^+^ cells	Increase in CD34^+^ B-lymphoid progenitors	[70]
Klinefelter syndrome (KS)	Transcriptome	Elucidate the molecular basis of infertility	Testis	Subpopulation of Sertoli cells in KS lack transcription from the XIST locus	[71]
Klinefelter syndrome (KS)	Transcriptome	Elucidate the molecular basis of the KS phenotype	Peripheral blood mononuclear cells	Identified candidate genes leading to KS phenotype	[72]
Systemic and mosaic aneuploidy (T21, T18, T8, T13)	Transcriptome	Molecular basis phenotype from copy number alterations	Skin; fibroblasts	In trisomic cells, the additional allele is independently transcribed	[73]
Trisomy 21 – Down syndrome	Transcriptome	Gene changes in cells with age	Brain	Microglial activation, increased inhibitory neurons, RNAs with intra-exonic junctions	[60]
Trisomy 18 – Edward’s syndrome	Chromatin accessibility	Disease mechanism and phenotype	Umbilical cord blood	Altered cell populations and misregulated pathways	[74]
Rett syndrome	Transcriptome	Gene expression in mosaic Rett syndrome	Occipital cortex	The upregulated genes in Rett syndrome are correlated with the extent of DNA methylation	[75]

The nuclear protein MECP2 is suggested to act as a transcriptional repressor by recruiting repressor elements to methylated DNA in a cell type-specific manner, a function which is impaired in the mutated MECP lacking the transcriptional repressor domain. As a result of the mosaicism, the neural circuits in female individuals with Rett syndrome are composed of normal as well as diseased neurons. Many of the previous investigations on the function of MECP2 were carried out on hemizygous male mice, where all the cells are affected, limiting the conclusions for mosaic states. Renthal et al. [75] addressed this by applying sc-transcriptome-seq as well as clever genotyping methods to establish genotype–phenotype relationships. However, one of the technical challenges in addressing a genotype–phenotype problem is that sc-transcriptome-seq technologies are not ideal for genotyping, because of several reasons: (1) they only detect variants in the transcribed regions; (2) most technologies (except for Smart-seq or other bespoke methods [60]) are designed for counting the transcripts as opposed to genotyping and therefore do not cover the full length of the transcript; and (3) they only capture a small fraction of the cellular transcriptome (usually 2000–3000 transcripts per cell). The authors therefore relied on identifying the cells expressing the wild-type allele or the mutant allele by taking advantage of allele-specific SNPs that were maintained in *cis* with the mutant *MECP2* gene. As a result, they were able to identify the ∼ 3000 dysregulated genes in excitatory neurons and ∼ 200 genes in vasoactive intestinal peptide interneurons. By taking advantage of published single-cell methylation data from the human cortex [76], they were able to conclude that indeed MECP2 in humans represses highly methylated long genes in wild-type but not *MECP2* mutant neurons, thus providing mechanistic insight into this disease.

**Box 2.**

**Rett syndrome.** X-linked genetic disorders, such as Rett syndrome, which is caused by mutations in the *MECP2* gene, or Fragile X syndrome, which is caused by repeat expansion within the *FMR1* gene [61], result in neurodevelopmental disorders. In female patients the manifestation of the disorder is generally milder as a result of mosaicism, where the affected tissues are composed of cells expressing either normal or non-functional genes due to the random inactivation of the alleles. Bulk/tissue-level sequencing methods are insensitive to the phenotypic characterization in these cases due to the lack of cellular resolution to tease apart somatic mosaicism. Sc-seq offers unique advantages. Not only does it offer single-cell resolution to characterize the cellular phenotypes, it also offers the possibility to compare the phenotypes between cells expressing the functional or the mutant *MECP2* obtained from the same individual, thus avoiding batch effects and biological variabilities when comparing Rett samples and age-matched controls. Moreover, since the cells with altered transcriptome in mosaic diseases are surrounded by an otherwise “normally functioning” environment, inter-cellular factors affecting the cells are also alleviated.

### ii. Pre-clinical applications to aid diagnostics and therapy

The ability to detect rare cellular subpopulations within a tissue biopsy has been the long-held promise of sc-seq technologies for early disease diagnosis, enabling timely therapeutic interventions (Fig. 2B). But, unlike the rapidly accelerating literature on such prospective applications of sc-seq for disease diagnosis [77], its explicit use in the clinic is lagging behind. Currently there are a disproportionately large number of reviews highlighting the promise of sc-seq and its diverse use case scenarios for diagnostics, therapeutic monitoring, and personalized medicine, especially in the context of cancer [78], [79], [80], [81], [82], [83], [84]. While several hurdles, as discussed in the Technological limitations section, prevent the direct application of sc-seq in the clinic, its usage, e. g., in clinical trials, to assess the (in)effectiveness of a new therapy and to delineate the underlying mechanisms has seen some interest [85], [86], [87].

One of the primary applications of the technology in a therapeutic setting has been in assessing and understanding the response (or resistance) to cancer therapy. For example, Kim et al. [88] used sc-seq on triple-negative breast cancer biopsies/excised samples to address an unresolved question, i. e., whether resistance to neoadjuvant chemotherapy was caused by generation of new mutations during the therapy or the selection of rare, pre-existing clones, owing to intra-tumor heterogeneity. By the combined use of sc-genome-seq and sc-transcriptome-seq the authors found out that while the chemotherapy caused the adaptive selection of pre-existing mutations, the selected cells underwent transcriptional reprogramming in response to the therapy.

Mochizuki et al. [89] report on their intermediate results from a Phase I clinical trial utilizing chimeric antigen receptor T-cell (CART) therapy for gliomas. They find correlations between immunosuppressive myeloid populations and the response to therapy based on sc-transcriptome-seq of cerebrospinal fluid biopsies. In this context, it is worth highlighting that the use of circulating cells, such as circulating tumor cells and cells in the cerebrospinal fluid, in combination with sc-seq is being recognized as a promising strategy for diagnosis and therapeutic monitoring [83]. Indeed tools specifically designed to enrich cells from patient biopsies with liquid samples are also being developed [90].

### iii. Applications under active development

Most aspects of sc-seq are yet to be standardized for routine clinical use. One of the reasons for this steady and continuous development is to build upon the basic idea of sequencing every cell and to introduce new capabilities. Incorporation of new capabilities constitutes one of the focus areas of the development, which include simultaneous recording of spatial characteristics with “sci-space” (see Box 3), longitudinal sequencing of the transcriptome in live cells with “live-seq” [91], or the recording of information related to cell physiology with “patch-seq” [92]. Another direction into which the technology is currently developing is to address fundamental and long-standing questions in biology, such as annotating the non-coding genome or unraveling the complexity of human development. These challenges are being addressed using novel approaches utilizing sc-seq such as “pooled CRISPR screening” [93] and “cell lineage tracing” [94], [95]. While not all of these breakthroughs are of immediate relevance to human genetics, we believe some of the outcomes of this global effort mandate a discussion for this readership, even if their benefits in the clinic may take years to fruition. These include: the construction of open-access cell atlases, the unbiased annotation of functional elements in the genome, and the high-throughput phenotyping of variants. We limit our discussion below to the cell atlases and the phenotyping of variants, since the annotation of functional elements in the genome was recently reviewed elsewhere [10].

**Figure 3:**
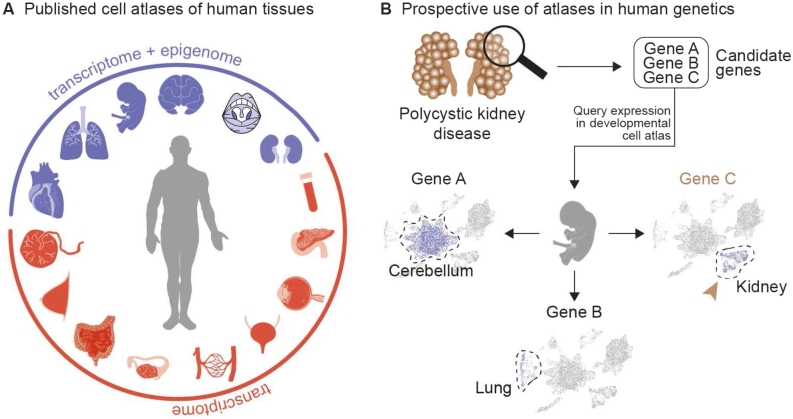
Wild type atlases of human tissues and their applications in human genetics diagnostics. **(A)** Single-cell atlases of many human organs and tissues are publicly available, some of which are highlighted here. Blue and red colors indicate the modality of sc-seq included in the dataset. Detailed information related to these atlases can be found in Supplementary Table 1. **(B)** Schematic of a use case scenario for the diagnosis of polycystic kidney disease, depicting the identification of candidate genes using a standard exome diagnostics workflow. This gene list can be further filtered based on expression analysis in the relevant (e. g., whole embryo or kidney) publicly available cell atlases. In the portrayed scenario, Gene C, expressed in the affected organ (brown arrowhead), will be prioritized for diagnostics. Note: Synthesized data.

**Box 3.**

**A word on spatial transcriptomics.** One of the drawbacks of sc-seq is the loss of spatial information when dissociating the tissue into cells. This loss is most noticed when an entire organism (e. g., whole embryos [23], [96] or zebrafish [97]) or a large tissue (e. g., brain [98]) is sequenced, where the spatial context is at least as important as the cell type information because of abundant cell types such as mesenchymal or epithelial cells. Spatial transcriptomics is a related sequencing methodology that, until recently, prioritized preserving spatial coordinates over cellular identity of the sequenced molecules [99]. Methods have been developed to integrate, experimentally [100] and bioinformatically [5], [101], the sc-seq data with spatial data, which can help delineate how tissue-level phenotypes form by collective cellular functions.

#### iii (a) Human and mouse cell atlases

Creating publicly accessible cell atlases of organisms based on sc-seq has been one focus area of the developmental research field (Fig. 3A). In a cell atlas, cells are classified and catalogued based on their expression profiles and epigenetic marks. They are an especially valuable resource for developmental and disease research, as they provide an open and peer-reviewed benchmark to compare diseased cellular states as well as to screen gene expression patterns or epigenetic modifications at a particular cell type, developmental time, or tissue (Fig. 3A). An example of this was discussed earlier – the work on Rett syndrome (Box 2). The international effort of building such cell atlases has seen contributions from individual labs through to one enormous single-cell experiment as well as international consortia, such as the Human Cell Atlas (https://www.humancellatlas.org/), collaborating to put together different sc-seq experiments to scan the whole body. The outcome has not only been extensive knowledge on how the cells work in an orchestrated fashion leading to the development of a functional organism, but it has also laid the groundwork to interpret diseased cellular states, which from a clinical perspective can aid early disease detection and therapy (Fig. 2). In a case example where the clinician aims to determine the phenotypic consequences of a deleterious frame-shift mutation found in a patient with a rare disease, where the expression pattern of the gene is still unknown, it will no longer require painstaking expression profiling (e. g., *in situ* hybridization assays). Instead, this information is readily available at the fingertips through web interfaces to atlases such as https://www.cambridgecellatlas.org/ or https://descartes.brotmanbaty.org/ (Fig. 3B). Whole-embryo atlases of other model organisms such as mice, including during its embryonic development between E9.5 and E13.5 [23] with spatial information [102], and those featuring pleiotropic mutations are also available [103]. Indeed new cell atlases are being published on a daily basis and it will take a concerted effort to collect, host, organize, and present this information in a useful, comparable, and seamless fashion to maximize their clinical benefits [104].

#### iii (b) Multiplexed, high-throughput phenotyping of variants

Current pipelines for the diagnosis of rare genetic diseases critically rely on functionally annotated variants. However, only about 30 % of the known variants in ClinVar have been definitely classified to be pathogenic or not, with nearly half the variants classified to be variants of uncertain significance (VUS). Indeed, the lack or uncertainty of some of the annotations has been attributed to the 10 % of the undiagnosed cases after whole-genome sequencing [1]. With whole-genome sequencing being envisaged as standard care and the increasing number of un-annotated variants being identified in coding and non-coding regions, there is an urgent need for high-throughput genotype–phenotype screening technologies that can establish (cf. bioinformatically predict) deleteriousness and phenotypic consequences of variants. The advent of approaches such as saturation genome editing (see accompanying article by Findlay et al. in this issue) have enabled the high-throughput generation and screening of thousands of coding and non-coding variants, as opposed to relying on population screens to identify variants, where the rate of discovery of rare variants is inherently limited by mutation rates and selective pressures. These approaches have also been beautifully and comprehensively reviewed elsewhere [93]. However, the throughput of genotyping has not yet been met in phenotyping, which is mostly limited to specific functional screens, by means of guide RNA representation in the population [105], application of selective pressure, or FACS sorting [106]. Sc-seq promises to provide this tool, offering unbiased, multiplexed, and high-throughput phenotyping capabilities. It has already found applications in the annotation of genomic regions [107], [108], [109], [110]. The combination of genome editing approaches with sc-seq technologies will eventually enable testing of all observed variants of a patient in one multiplexed experiment. This will have an immediate impact on human genetics and help to establish genotype–phenotype relationships for variants across the entire human genome at scale.

## Technological limitations

Sc-seq shares some of the fundamental challenges with bulk-seq in capturing and amplifying the nucleic acid from the samples, leading to PCR amplification biases, dropout events, and allelic imbalance, except that these biases are more exaggerated in sc-seq due to the limited nucleic acid content in individual cells. Bioinformatic quality control tools currently represent the primary strategy used to tackle such experimental artifacts. For example, tools such as Scrublet [111] have been developed to detect doublet cells in sc-seq to be filtered out, which may otherwise corrode the data. Appropriate extraction and handling of the samples are also vital, since the qualities of chromatin and the RNA are known to have a direct influence on the data quality [112]. Current sc-seq sequencing methods also face a real trade-off between sequencing coverage and number of cells sequenced [10]. Commercially available kits such as 10x Genomics toolkits help sequence 20,000 cells and detect a few thousand genes per cell, running the risk of smaller cell populations or phenotypes with subtle gene expression changes left undetected. However, advanced bioinformatic tools can help overcome some of these experimental limitations, as demonstrated by us and others by detecting even minor changes in both gene expression and cell type compositions in mutant mouse embryos [103].

## Conclusions

In basic research, sc-seq technologies have been widely established as a toolbox to query developmental processes and disease mechanisms with unprecedented sensitivity and granularity. Despite neck-breaking advances over the last decade, the technology is in many ways still nascent. That is, the data created and conclusions drawn do not yet suffice as a “one-experiment proof” and continue to require reinforcements with additional validations. There are indications of increased transfer of sc-seq to a number of fields, including human genetics, with tens of genetic diseases that have already been characterized with this technology (Table 1). With sequencing and library preparation costs rapidly dropping, the protocols being standardized [113], and bioinformatic tools becoming more accessible, the barriers to translation are rapidly vanishing. The ultimate promise of the technology for the field of human genetics is to offer the means for massively parallel functional variants testing *in vitro* and at some stage also *in vivo*. While we are not there yet, we expect the technology to be ripe for adoption within the present decade. Education will ultimately play a key role in realizing this and we hope this review has contributed towards informing this readership of human geneticists about the current state of this recent and booming technology.

## Supplementary Material

A list of publicly available single-cell atlases of human organs and whole embryos.

## References

[1] 100,000 Genomes Project Pilot Investigators, Smedley D, Smith KR (2021). 100,000 Genomes Pilot on Rare-Disease Diagnosis in Health Care – Preliminary Report. N Engl J Med.

[2] Rajewsky N, Almouzni G, Gorski SA (2020). LifeTime and improving European healthcare through cell-based interceptive medicine. Nature.

[3] Elmentaite R, Domínguez Conde C, Yang L, Teichmann SA (2022). Single-cell atlases: shared and tissue-specific cell types across human organs. Nat Rev Genet.

[4] Gawad C, Koh W, Quake SR (2016). Single-cell genome sequencing: current state of the science. Nat Rev Genet.

[5] Longo SK, Guo MG, Ji AL, Khavari PA (2021). Integrating single-cell and spatial transcriptomics to elucidate intercellular tissue dynamics. Nat Rev Genet.

[6] Teschendorff AE, Feinberg AP (2021). Statistical mechanics meets single-cell biology. Nat Rev Genet.

[7] Andrews TS, Kiselev VY, McCarthy D, Hemberg M (2021). Tutorial: guidelines for the computational analysis of single-cell RNA sequencing data. Nat Protoc.

[8] Haque A, Engel J, Teichmann SA, Lönnberg T (2017). A practical guide to single-cell RNA-sequencing for biomedical research and clinical applications. Genome Med.

[9] Luecken MD, Theis FJ (2019). Current best practices in single-cell RNA-seq analysis: a tutorial. Mol Syst Biol.

[10] Sreenivasan VKA, Balachandran S, Spielmann M (2022). The role of single-cell genomics in human genetics. J Med Genet.

[11] Lodato MA, Woodworth MB, Lee S (2015). Somatic mutation in single human neurons tracks developmental and transcriptional history. Science.

[12] Evrony GD, Hinch AG, Luo C (2021). Applications of Single-Cell DNA Sequencing. Annu Rev Genomics Hum Genet.

[13] Cao J, Cusanovich DA, Ramani V (2018). Joint profiling of chromatin accessibility and gene expression in thousands of single cells. Science.

[14] Chen S, Lake BB, Zhang K (2019). High-throughput sequencing of the transcriptome and chromatin accessibility in the same cell. Nat Biotechnol.

[15] Gierahn TM, Wadsworth MH, Hughes TK (2017). Seq-Well: portable, low-cost RNA sequencing of single cells at high throughput. Nat Methods.

[16] Yuan J, Sims PA (2016). An Automated Microwell Platform for Large-Scale Single Cell RNA-Seq. Sci Rep.

[17] Klein AM, Mazutis L, Akartuna I (2015). Droplet barcoding for single-cell transcriptomics applied to embryonic stem cells. Cell.

[18] Macosko EZ, Basu A, Satija R (2015). Highly Parallel Genome-wide Expression Profiling of Individual Cells Using Nanoliter Droplets. Cell.

[19] Yin Y, Jiang Y, Lam K-WG (2019). High-Throughput Single-Cell Sequencing with Linear Amplification. Mol Cell.

[20] Ashton JM, Rehrauer H, Myers J (2021). Comparative Analysis of Single-Cell RNA Sequencing Platforms and Methods. J Biomol Tech.

[21] Ziegenhain C, Vieth B, Parekh S (2017). Comparative Analysis of Single-Cell RNA Sequencing Methods. Mol Cell.

[22] Simmons SK, Lithwick-Yanai G, Adiconis X (2022). Mostly natural sequencing-by-synthesis for scRNA-seq using Ultima sequencing. Nat Biotechnol.

[23] Cao J, Spielmann M, Qiu X (2019). The single-cell transcriptional landscape of mammalian organogenesis. Nature.

[24] Galdos FX, Xu S, Goodyer WR (2022). devCellPy is a machine learning-enabled pipeline for automated annotation of complex multilayered single-cell transcriptomic data. Nat Commun.

[25] Zappia L, Theis FJ (2021). Over 1000 tools reveal trends in the single-cell RNA-seq analysis landscape. Genome Biol.

[26] Chen H, Lareau C, Andreani T (2019). Assessment of computational methods for the analysis of single-cell ATAC-seq data. Genome Biol.

[27] Valecha M, Posada D (2022). Somatic variant calling from single-cell DNA sequencing data. Comput Struct Biotechnol J.

[28] Gardeux V, David FPA, Shajkofci A (2017). ASAP: a web-based platform for the analysis and interactive visualization of single-cell RNA-seq data. Bioinformatics.

[29] Ianevski A, Giri AK, Aittokallio T (2022). Fully-automated and ultra-fast cell-type identification using specific marker combinations from single-cell transcriptomic data. Nat Commun.

[30] Huang W, Wang D, Yao Y-F (2021). Understanding the pathogenesis of infectious diseases by single-cell RNA sequencing. Microb Cell Fact.

[31] Potter SS (2018). Single-cell RNA sequencing for the study of development, physiology and disease. Nat Rev Nephrol.

[32] Lei Y, Tang R, Xu J (2021). Applications of single-cell sequencing in cancer research: progress and perspectives. J Hematol Oncol.

[33] Li Y, Polyak D, Lamsam L (2021). Comprehensive RNA analysis of CSF reveals a role for CEACAM6 in lung cancer leptomeningeal metastases. NPJ Precis Oncol.

[34] Miles LA, Bowman RL, Merlinsky TR (2020). Single-cell mutation analysis of clonal evolution in myeloid malignancies. Nature.

[35] Navin N, Kendall J, Troge J (2011). Tumour evolution inferred by single-cell sequencing. Nature.

[36] Ruan H, Wang Z, Sun Z (2022). Single-cell RNA sequencing reveals the characteristics of cerebrospinal fluid tumour environment in breast cancer and lung cancer leptomeningeal metastases. Clin Transl Med.

[37] D’Gama AM, Walsh CA (2018). Somatic mosaicism and neurodevelopmental disease. Nat Neurosci.

[38] Samad T, Wu SM (2021). Single cell RNA sequencing approaches to cardiac development and congenital heart disease. Semin Cell Dev Biol.

[39] Iskander D, Wang G, Heuston EF (2021). Single-cell profiling of human bone marrow progenitors reveals mechanisms of failing erythropoiesis in Diamond-Blackfan anemia. Sci Transl Med.

[40] Velmeshev D, Schirmer L, Jung D (2019). Single-cell genomics identifies cell type-specific molecular changes in autism. Science.

[41] Der E, Ranabothu S, Suryawanshi H (2017). Single cell RNA sequencing to dissect the molecular heterogeneity in lupus nephritis. JCI Insight.

[42] Schafflick D, Xu CA, Hartlehnert M (2020). Integrated single cell analysis of blood and cerebrospinal fluid leukocytes in multiple sclerosis. Nat Commun.

[43] Stephenson W, Donlin LT, Butler A (2018). Single-cell RNA-seq of rheumatoid arthritis synovial tissue using low-cost microfluidic instrumentation. Nat Commun.

[44] Jackson ND, Everman JL, Chioccioli M (2020). Single-Cell and Population Transcriptomics Reveal Pan-epithelial Remodeling in Type 2-High Asthma. Cell Rep.

[45] Li H, Wang H, Sokulsky L (2021). Single-cell transcriptomic analysis reveals key immune cell phenotypes in the lungs of patients with asthma exacerbation. J Allergy Clin Immunol.

[46] Vieira Braga FA, Kar G, Berg M (2019). A cellular census of human lungs identifies novel cell states in health and in asthma. Nat Med.

[47] Lodato MA, Rodin RE, Bohrson CL (2018). Aging and neurodegeneration are associated with increased mutations in single human neurons. Science.

[48] Miller MB, Reed HC, Walsh CA (2021). Brain Somatic Mutation in Aging and Alzheimer’s Disease. Annu Rev Genomics Hum Genet.

[49] Menon M, Mohammadi S, Davila-Velderrain J (2019). Single-cell transcriptomic atlas of the human retina identifies cell types associated with age-related macular degeneration. Nat Commun.

[50] Voigt AP, Mulfaul K, Mullin NK (2019). Single-cell transcriptomics of the human retinal pigment epithelium and choroid in health and macular degeneration. Proc Natl Acad Sci USA.

[51] Wang Z, Su D, Liu S (2021). RNA sequencing and bioinformatics analysis of human lens epithelial cells in age-related cataract. BMC Ophthalmol.

[52] Jiang J, Wang C, Qi R (2020). scREAD: A Single-Cell RNA-Seq Database for Alzheimer’s Disease. iScience.

[53] Wang M, Song W-M, Ming C (2022). Guidelines for bioinformatics of single-cell sequencing data analysis in Alzheimer’s disease: review, recommendation, implementation and application. Mol Neurodegener.

[54] Kamath T, Abdulraouf A, Burris SJ (2022). Single-cell genomic profiling of human dopamine neurons identifies a population that selectively degenerates in Parkinson’s disease. Nat Neurosci.

[55] Smajić S, Prada-Medina CA, Landoulsi Z (2022). Single-cell sequencing of human midbrain reveals glial activation and a Parkinson-specific neuronal state. Brain.

[56] Wang Q, Wang M, Choi I, et al. Single-cell transcriptomic atlas of the human substantia nigra in Parkinson’s disease. bioRxiv. 2022. https://doi.org/10.1101/2022.03.25.485846..

[57] Ahmadi A, Gispert JD, Navarro A, Vilor-Tejedor N, Sadeghi I (2021). Single-cell Transcriptional Changes in Neurodegenerative Diseases. Neuroscience.

[58] Godfrey M, Lee NR (2018). Memory profiles in Down syndrome across development: a review of memory abilities through the lifespan. J Neurodev Disord.

[59] Wisniewski KE, Wisniewski HM, Wen GY (1985). Occurrence of neuropathological changes and dementia of Alzheimer’s disease in Down’s syndrome. Ann Neurol.

[60] Palmer CR, Liu CS, Romanow WJ (2021). Altered cell and RNA isoform diversity in aging Down syndrome brains. Proc Natl Acad Sci USA.

[61] Bagni C, Tassone F, Neri G, Hagerman R (2012). Fragile X syndrome: causes, diagnosis, mechanisms, and therapeutics. J Clin Invest.

[62] Li Q, Wang Y, Deng W (2021). Heterogeneity of cell composition and origin identified by single-cell transcriptomics in renal cysts of patients with autosomal dominant polycystic kidney disease. Theranostics.

[63] Muto Y, Dixon EE, Yoshimura Y, et al. Defining cellular complexity in human autosomal dominant polycystic kidney disease by multimodal single cell analysis. bioRxiv. 2021. https://doi.org/10.1101/2021.10.21.465323..

[64] Al-Dalahmah O, Sosunov AA, Shaik A (2020). Single-nucleus RNA-seq identifies Huntington disease astrocyte states. Acta Neuropathol Commun.

[65] Garcia FJ, Sun N, Lee H (2022). Single-cell dissection of the human brain vasculature. Nature.

[66] Lee H, Fenster RJ, Pineda SS (2020). Cell Type-Specific Transcriptomics Reveals that Mutant Huntingtin Leads to Mitochondrial RNA Release and Neuronal Innate Immune Activation. Neuron.

[67] Schupp JC, Khanal S, Gomez JL (2020). Single-Cell Transcriptional Archetypes of Airway Inflammation in Cystic Fibrosis. Am J Respir Crit Care Med.

[68] Carraro G, Langerman J, Sabri S (2021). Transcriptional analysis of cystic fibrosis airways at single-cell resolution reveals altered epithelial cell states and composition. Nat Med.

[69] Valenzi E, Bulik M, Tabib T (2019). Single-cell analysis reveals fibroblast heterogeneity and myofibroblasts in systemic sclerosis-associated interstitial lung disease. Ann Rheum Dis.

[70] Hua P, Roy N, de la Fuente J (2019). Single-cell analysis of bone marrow–derived CD34+ cells from children with sickle cell disease and thalassemia. Blood.

[71] Mahyari E, Guo J, Lima AC (2021). Comparative single-cell analysis of biopsies clarifies pathogenic mechanisms in Klinefelter syndrome. Am J Hum Genet.

[72] Liu X, Tang D, Zheng F (2019). Single-Cell Sequencing Reveals the Relationship between Phenotypes and Genotypes of Klinefelter Syndrome. Cytogenet Genome Res.

[73] Stamoulis G, Garieri M, Makrythanasis P (2019). Single cell transcriptome in aneuploidies reveals mechanisms of gene dosage imbalance. Nat Commun.

[74] Qiu X, Yu H, Wu H (2021). Single-cell chromatin accessibility landscape of human umbilical cord blood in trisomy 18 syndrome. Hum Genomics.

[75] Renthal W, Boxer LD, Hrvatin S (2018). Characterization of human mosaic Rett syndrome brain tissue by single-nucleus RNA sequencing. Nat Neurosci.

[76] Luo C, Keown CL, Kurihara L (2017). Single-cell methylomes identify neuronal subtypes and regulatory elements in mammalian cortex. Science.

[77] Kim N, Kim HK, Lee K (2020). Single-cell RNA sequencing demonstrates the molecular and cellular reprogramming of metastatic lung adenocarcinoma. Nat Commun.

[78] Deng Z, Wu S, Wang Y, Shi D (2022). Circulating tumor cell isolation for cancer diagnosis and prognosis. EBioMedicine.

[79] Kamies R, Martinez-Jimenez CP (2020). Advances of single-cell genomics and epigenomics in human disease: where are we now?. Mamm Genome.

[80] Mustachio LM, Roszik J (2022). Single-Cell Sequencing: Current Applications in Precision Onco-Genomics and Cancer Therapeutics. Cancers.

[81] Sklavenitis-Pistofidis R, Getz G, Ghobrial I (2021). Single-cell RNA sequencing: one step closer to the clinic. Nat Med.

[82] Tang X, Huang Y, Lei J (2019). The single-cell sequencing: new developments and medical applications. Cell Biosci.

[83] Yekula A, Tracz J, Rincon-Torroella J (2022). Single-Cell RNA Sequencing of Cerebrospinal Fluid as an Advanced Form of Liquid Biopsy for Neurological Disorders. Brain Sci.

[84] Zhu W, Zhang X-Y, Marjani SL (2017). Next-generation molecular diagnosis: single-cell sequencing from bench to bedside. Cell Mol Life Sci.

[85] Cohen YC, Zada M, Wang S-Y (2021). Identification of resistance pathways and therapeutic targets in relapsed multiple myeloma patients through single-cell sequencing. Nat Med.

[86] Samur MK, Fulciniti M, Aktas Samur A (2021). Biallelic loss of BCMA as a resistance mechanism to CAR T cell therapy in a patient with multiple myeloma. Nat Commun.

[87] Wu H, Malone AF, Donnelly EL (2018). Single-Cell Transcriptomics of a Human Kidney Allograft Biopsy Specimen Defines a Diverse Inflammatory Response. J Am Soc Nephrol.

[88] Kim C, Gao R, Sei E (2018). Chemoresistance Evolution in Triple-Negative Breast Cancer Delineated by Single-Cell Sequencing. Cell.

[89] Mochizuki A, Ramakrishna S, Good Z (2021). Omic-11. Single cell RNA sequencing from the CSF of subjects with H3k27M+ DIPG/DMG treated with GD2 CAR T-cellular therapy. Neuro-Oncol.

[90] Cheng Y-H, Chen Y-C, Lin E (2019). Hydro-Seq enables contamination-free high-throughput single-cell RNA-sequencing for circulating tumor cells. Nat Commun.

[91] Chen W, Guillaume-Gentil O, Rainer PY (2022). Live-seq enables temporal transcriptomic recording of single cells. Nature.

[92] Cadwell CR, Palasantza A, Jiang X (2016). Electrophysiological, transcriptomic and morphologic profiling of single neurons using Patch-seq. Nat Biotechnol.

[93] Bock C, Datlinger P, Chardon F (2022). High-content CRISPR screening. Nat Rev Methods Primers.

[94] Choi J, Chen W, Minkina A (2022). A time-resolved, multi-symbol molecular recorder via sequential genome editing. Nature.

[95] Kong W, Biddy BA, Kamimoto K (2020). CellTagging: combinatorial indexing to simultaneously map lineage and identity at single-cell resolution. Nat Protoc.

[96] Cao J, O’Day DR, Pliner HA (2020). A human cell atlas of fetal gene expression. Science.

[97] Farrell JA, Wang Y, Riesenfeld SJ (2018). Single-cell reconstruction of developmental trajectories during zebrafish embryogenesis. Science.

[98] Eze UC, Bhaduri A, Haeussler M (2021). Single-cell atlas of early human brain development highlights heterogeneity of human neuroepithelial cells and early radial glia. Nat Neurosci.

[99] Ståhl PL, Salmén F, Vickovic S (2016). Visualization and analysis of gene expression in tissue sections by spatial transcriptomics. Science.

[100] Srivatsan SR, Regier MC, Barkan E (2021). Embryo-scale, single-cell spatial transcriptomics. Science.

[101] Biancalani T, Scalia G, Buffoni L (2021). Deep learning and alignment of spatially resolved single-cell transcriptomes with Tangram. Nat Methods.

[102] Chen A, Liao S, Cheng M (2022). Spatiotemporal transcriptomic atlas of mouse organogenesis using DNA nanoball-patterned arrays. Cell.

[103] Huang X, Henck J, Qiu C, et al. Single cell, whole embryo phenotyping of pleiotropic disorders of mammalian development. bioRxiv. 2022. https://doi.org/10.1101/2022.08.03.500325..

[104] Aevermann BD, Novotny M, Bakken T (2018). Cell type discovery using single-cell transcriptomics: implications for ontological representation. Hum Mol Genet.

[105] Findlay GM, Boyle EA, Hause RJ (2014). Saturation editing of genomic regions by multiplex homology-directed repair. Nature.

[106] Erwood S, Bily TMI, Lequyer J (2022). Saturation variant interpretation using CRISPR prime editing. Nat Biotechnol.

[107] Adamson B, Norman TM, Jost M (2016). A Multiplexed Single-Cell CRISPR Screening Platform Enables Systematic Dissection of the Unfolded Protein Response. Cell.

[108] Datlinger P, Rendeiro AF, Schmidl C (2017). Pooled CRISPR screening with single-cell transcriptome readout. Nat Methods.

[109] Gasperini M, Hill AJ, McFaline-Figueroa JL (2019). A Genome-wide Framework for Mapping Gene Regulation via Cellular Genetic Screens. Cell.

[110] Jaitin DA, Weiner A, Yofe I (2016). Dissecting Immune Circuits by Linking CRISPR-Pooled Screens with Single-Cell RNA-Seq. Cell.

[111] Wolock SL, Lopez R, Klein AM (2019). Scrublet: Computational Identification of Cell Doublets in Single-Cell Transcriptomic Data. Cell Syst.

[112] Lafzi A, Moutinho C, Picelli S, Heyn H (2018). Tutorial: guidelines for the experimental design of single-cell RNA sequencing studies. Nat Protoc.

[113] Mereu E, Lafzi A, Moutinho C (2020). Benchmarking single-cell RNA-sequencing protocols for cell atlas projects. Nat Biotechnol.

